# Contrasting seasonal niche separation between rare and abundant taxa conceals the extent of protist diversity

**DOI:** 10.1111/j.1365-294X.2010.04669.x

**Published:** 2010-07

**Authors:** VIOLA NOLTE, RAM VINAY PANDEY, STEFFEN JOST, RALPH MEDINGER, BIRGIT OTTENWÄLDER, JENS BOENIGK, CHRISTIAN SCHLÖTTERER

**Affiliations:** * Institut für Populationsgenetik, Veterinärmedizinische Universität WienVeterinärplatz 1, A-1210 Vienna, Austria; † Austrian Academy of Sciences, Institute for Limnolgy, Herzog Odilostraße 101A-5310 Mondsee, Austria; ‡ University of Innsbruck, Faculty of BiologyTechnikerstraße 15, A-6020 Innsbruck, Austria; § Eurofins Medigenomics GmbHAnzingerstr. 7a, D-85560 Ebersberg, Germany

**Keywords:** 2nd generation sequencing, 454 protist diversity, species turnover, temporal sampling

## Abstract

With the advent of molecular methods, it became clear that microbial biodiversity had been vastly underestimated. Since then, species abundance patterns were determined for several environments, but temporal changes in species composition were not studied to the same level of resolution. Using massively parallel sequencing on the 454 GS FLX platform we identified a highly dynamic turnover of the seasonal abundance of protists in the Austrian lake Fuschlsee. We show that seasonal abundance patterns of protists closely match their biogeographic distribution. The stable predominance of few highly abundant taxa, which previously led to the suggestion of a low global protist species richness, is contrasted by a highly dynamic turnover of rare species. We suggest that differential seasonality of rare and abundant protist taxa explains the—so far—conflicting evidence in the ‘everything is everywhere’ dispute. Consequently temporal sampling is basic for adequate diversity and species richness estimates.

## Introduction

Molecular biology has filled in some of the blanks about the natural history of microbial plankton, disclosing that four out of six eukaryotic kingdoms largely comprise protists (Cavalier-Smith 1998; Adl *et al.* 2005). Cultivation independent environmental surveys demonstrated an underestimation of protist diversity by orders of magnitude (Diez *et al.* 2001; Lopez-Garcia *et al.* 2001; Moon-van der Staay *et al.* 2001) and stimulated protist diversity research within the last decade. Although molecular data have revealed the vast scope of microbial diversity in virtually all habitats (Green *et al.* 2004; Slapeta *et al.* 2005), the fundamental conflict between ubiquitous and endemic distribution patterns, as basic to the ‘everything is everywhere’ dispute (Finlay 2002; Telford *et al.* 2006; Foissner 2007; Cermeno & Falkowski 2009), is far from being solved.

Two main hypotheses about the causes of non-random distribution of micro-organisms continue to obtain conflicting experimental support: the idea that ‘everything is everywhere’ (Beijerinck 1913; Baas-Becking 1934) is based on the assumption that the enormous dispersal capabilities of micro-organisms allow them to spread into virtually any habitat (Finlay 2002). Absence of taxa is caused by unfavourable local conditions that prevent them from getting established everywhere (Cermeno & Falkowski 2009). In a contrasting model, the rate of dispersal of micro-organisms is not sufficiently high to overcome historical dispersal limitations and human influence. This allows for existence of endemic taxa many of which remain to be discovered (Telford *et al.* 2006; Foissner 2007).

The debate about the biogeography of micro-organisms is centred on spatial distribution patterns and most studies sample microbial assemblages at a single time point to assess whether relative abundances of taxa differ among habitats.

Despite that seasonal niche separation could lead to an omission of taxa in environmental surveys, seasonality of protists has insufficiently been addressed. Frequency shifts in the composition of protist communities were mostly studied at higher taxonomic levels (Tamigneaux *et al.* 1997; Araújo & Godinho 2008), in highly abundant taxa (Rynearson *et al.* 2006; Aguilera *et al.* 2007) or suffered from too shallow sequencing (Massana *et al.* 2004).

Massively parallel sequencing techniques now allow temporal surveys with sufficiently deep taxon sampling. However, high throughput sequencing approaches so far focused on phylogenetic relationships in protists (Burki *et al.* 2009) or species richness and spatial distribution (Amaral-Zettler *et al.* 2009; Stoeck *et al.* 2009) of protists largely ignoring the potential seasonality of protist community composition. Aquatic protists are present at millions of cells per litre and double every few hours to days. Extreme short-term variation of abundance pattern is therefore to be expected, but may differ between distinct groups of protists or habitats.

## Materials and methods

### Sampling and sample preparation

We collected 10 samples from the Lake Fuschlsee (Salzkammergut, Austria) in roughly 3-week intervals (March 2007–October 2007). Each temporal sample was collected at the same sampling site (47°48′26′′N, 13°15′34′′ E): integrated samples covering the upper 10 m of the water column were collected within the pelagic zone with a sampling tube. We pooled three integrated samples prior to further processing. Subsamples of 100 mL were filtered onto 0.2 μm polycarbonate filters for high-throughput sequencing. Filters were air dried and frozen at –80 °C until further processing.

### Sample preparation for 454 pyrosequencing

Genomic DNA was extracted with the DNeasy Tissue kit (Qiagen) for each temporal sample. Filters were transferred to a 2 mL tube and incubated for 1–3 h in buffer ATL of the DNeasy Tissue kit (Qiagen) supplemented with Proteinase K. DNA was subsequently extracted following the instructions of the supplier. We used HPLC-purified PCR primers, which carry sequences specific for the small subunit (SSU) of the rRNA gene (fw: ATTAGGGTTCGATTCCGGAGAGG, rv: CTGGAATTACCGCGGSTGCTG) and a 5′-tail for the 454 sequencing (adapter A: GCCTCCCTCGCGCCATCAG, adapter B: GCCTTGCCAGCCCGCTCAG). The forward primer also contained a 4 bp tag for each of the temporal samples inserted between the 454-adapter A and the SSU-specific part. The forward primer was based on the established broad eukaryotic primer Sogin2f (Auinger *et al.* 2008) while the reverse primer was newly designed. PCR primers were designed in conserved regions of the SSU rRNA gene to target eukaryotes. Hence, we aligned sequences from representative taxa covering all major freshwater protists. Primer specificity was tested by analysing clone libraries based on Sanger sequencing and later by analysis of the 454 dataset. The primers target all eukaryotic supergroups; some lineages may, however, not be targeted as it is generally known for broad eukaryotic primer sets (Stoeck *et al.* 2006). The primers amplify a 180–200 bp fragment of the SSU rRNA gene including the V3 region. PCR was carried out with 0.4U Phusion High-Fidelity DNA Polymerase (Finnzymes Oy, Finland), 200 μm dNTPs and 0.25 pmol of each primer. The cycling profile consisted of 1 min denaturation at 98 °C, followed 22 PCR cycles (98 °C for 10 s, 65 °C for 15 s, 72 °C for 20 s) with a final extension step of 7 min at 72°C.

In order to minimize recombinant PCR products, we only performed 22 PCR cycles as recommended by Qiu *et al.* (2001) and compensated for the lower yield by pooling the products of 10 independent PCRs. The pooled PCR products of each temporal sample were gel-purified using QIAquick Gel Extraction Kit (Qiagen, Hilden, Germany) and quantified on an agarose gel. We pooled 10 ng from each temporal sample and sequenced one test lane (for titration purpose) and one full picotiterplate on a 454 Roche FLX sequencer. Sequences have been deposited in GenBank (accession number SRA008706.3).

### Bioinformatic analyses

#### Adapter and primer clipping

The high rate of insertion–deletion errors inherent to the 454 pyrosequencing process required the development of an adapter and primer clipping software, which is specifically designed to account for these sequencing artefacts. We performed a pattern search to allow for mutations in homopolymers within the adapter B sequence during trimming but required the PCR primer sequences to be mutation free, otherwise the read was discarded. Reads for which an indel was identified at the transition to between read and PCR primer were removed. The detailed clipping algorithm of our CANGS package is described elsewhere (Pandey *et al.* 2010) and is available from http://i122server.vu-wien.ac.at/pop/software.html.

#### Quality filtering

In addition to primer clipping, we removed all sequences that did not fit the following criteria: (i) no Ns; (ii) quality score >24, when averaged across the read after clipping adapters and primers; (iii) minimum sequence length of 200 bp (including PCR primers); and (iv) at least two copies of the read present in the entire data set before clipping primers. This procedure eliminated approximately 37% of all sequences.

#### Taxonomic classification

In order to minimize the computational burden for genetic distance calculation and BLAST analysis, we first identified all non-redundant sequences and recorded their abundance in the complete as well as in each monthwise data set. In this step, we treated gaps as informative character and recorded sequences differing by a gap as two different non-redundant sequences. We blasted all non-redundant sequences against the NCBI database and retrieved the taxonomic classification of the best hit. In the case of multiple hits with identical E-values, we selected the most detailed taxonomic classification.

#### Analysis of taxon-specific variation

We used sequences affiliated with the *Spumella* morphotype to analyse taxon-specific temporal variation in abundance. The *Spumella* morphotype consists of colourless, non-scaled taxa within the C subcluster of the Chrysophyceae. Sequences belonging to the *Spumella* morphotype were identified by blasting all 454 sequences against the NCBI database. All sequences which were identical to or differed by a single base substitution (99.4% similarity) from known *Spumella*-sequences were included in the analysis.

#### Rarefaction analysis

We used the Analytic Rarefaction software (http://www.uga.edu/~strata/software/) for rarefaction calculations.

### Statistical analysis

Each monthwise sample was represented by a different number of sequence reads. In order to compare the abundance pattern between months we randomly selected the same number of sequences from each month (matching the sample with the lowest number of reads, i.e. 14 226 sequence reads after trimming and quality filtering). Sequence gaps were not considered, as they could either be a sequencing artefact or a biological variant. For the analysis of seasonal turnover we defined each non-redundant sequence as a separate OTU. We blasted each non-redundant sequence of one sample against all non-redundant sequences of each of the other samples and used a BLAST similarity cutoff of 100% to identify non-redundant sequences of a given month in each of the other months. The procedure is implemented in the CANGS software package described in Pandey *et al.* (2010). For each monthwise sample we determined the number of distinct OTUs (*i*), the frequency of each of these OTUs in other samples (*N*) and the number of reads for this OTU in the whole dataset (*n*). We further calculated the mean sequence abundance of the OTUs considering only samples in which these were present (*A*= *n*/*N*). Based on the 10 seasonal samples we calculated mean and SD for N and A for OTUs which were found in 1 (2, 3, …, 9,10) samples, respectively. Differences between the number of OTUs present in a different number of months were tested in a one way anova followed by Tukey’s Posthoc test (SigmaPlot 11).

Sequences with abundance >10 within the complete dataset were selected for a subsequent analysis of abundance heterogeneity between samples. For each sequence the counts in a given sample were log-transformed. These log-transformed counts were sorted in decreasing order and the slope m was calculated for each regression of the log-transformed counts vs. order rank. We subsequently calculated the correlation statistics for these slopes (*m*) as a function of the number of samples in which the respective sequence was observed using the SigmaPlot 11 software. The slope refers to the decrease in abundance between (ranked) samples and indicates therefore the strength of the shifts in abundance for the respective sequence. Error bars indicate the variation within a ranked sample.

## Results and discussion

### Richness of protist taxa estimated from single samples is unreliable due to seasonal variation

**Fig. 1:**
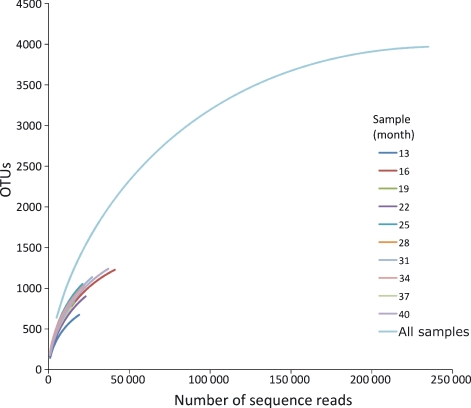
Rarefaction analysis of 10 seasonal samples from the Austrian lake Fuschlsee. The collection time for each sample is indicated by the week of collection and the combined data set is marked as ‘all samples’. While the OTU abundance estimate for all seasonal samples is very similar, the combined data set resulted in substantially higher estimates of OTU abundance.

Between March 2007 and October 2007 we collected 10 samples from Lake Fuschlsee in approximately 3 week intervals. Each sample covered the upper 10 m of the water column. We obtained 447 910 raw sequence reads of which, after stringent quality filtering, a total of 279 336 sequences could be analysed. In order to test the stability of protist community composition, we were first interested in differences between the taxonomic richness across seasons. We performed rarefaction analysis for every seasonal sample and found that in none of our samples the rarefaction curve showed full saturation. Interestingly, estimates of taxa richness for our rarefaction analysis were similar in all samples ranging from 673 to 1239 (Fig. 1). We repeated the rarefaction analysis using the pooled data set and obtained an approximately four-fold higher taxonomic diversity than from the analysis of a single month (Fig. 1). According to this analysis, the protist community does not only undergo quantitative fluctuations within a stable set of taxa that are present throughout the entire year. Instead, taxa present in 1 month can be entirely absent in the following and replaced by a different set of previously absent taxa. This analysis suggests that biodiversity is not adequately represented by a single sample; instead, to obtain reliable estimates of biodiversity in a given habitat it is important to cover the entire season.

### Seasonal succession reflects lineage-specific geographic centres of distribution

**Fig. 2:**
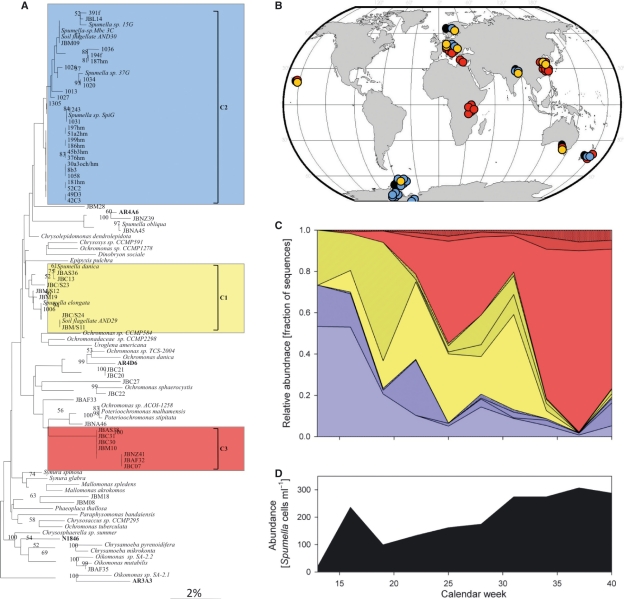
Spatial and seasonal distribution pattern of unpigmented flagellates affiliated with the *Spumella* morphotype (chrysophycean clusters C1, C2, C3). (A) Neighbour-joining tree showing the affiliation of 18S rRNA gene sequences from ‘*Spumella*-like’ isolates to the Chrysophyceae sensu stricto [modified from Findenig *et al.* (unpublished)]. The numbers at the nodes of the tree indicate percentage of bootstrap values for each node out of 100 bootstrap resamplings (values above 60 are shown). The scale bar indicates 2% estimated sequence divergence. Within the Chryophyceae *sensu stricto*, *Spumella*-like strains are affiliated with different subclusters and are most probably polyphyletic (Andersen *et al.* 1999; Boenigk *et al.* 2005). Three major different major taxonomic lineages of the *Spumella* morphotype can be distinguished within the chrysophyceaen C-cluster. Each of these lineages is indicated by a separate colour. (B) Biogeographic distribution of the three lineages based on isolated strains. Strains may occur also in other climatic zones but the observed pattern suggests different centres of distribution: the subcluster C2 (blue) comprises flagellates from different cold to temperate sites. The subcluster C1 includes mostly isolates originating from soil, mainly from temperate to moderately warm habitats. The subcluster C3 comprises exclusively freshwater isolates from temperate to warm habitats and (Boenigk *et al.* 2006). (C) Relative abundance pattern of the three major taxonomic lineages of the *Spumella* morphotype during the sampling period. All sequences previously described for the three clades were blasted against our 454 data, but only a subset was detected. Different shadings in each cluster indicate sequences that were detected in the 454 data set (either matching perfectly or differing by a single base pair difference). Sequences in the cluster with a frequency lower than 0.01% were grouped together. Species from cold environments were more abundant early in the year, while species from warmer environments were more frequent later in the year. Hence, the temporal abundance pattern matches the previously described biogeographic distribution. (D) Abundance of *Spumella*-like flagellates in Lake Fuschlsee based on microscopical analysis. Data of the microscopical analysis were taken from Medinger *et al.* (2010).

Despite similar estimates of taxon richness, the abundance pattern of individual taxa changed dramatically between samples. We focused on sequences affiliated with the *Spumella* morphospecies complex to analyse the seasonal turnover of their abundance. *Spumella* spp. are heterotrophic chrysophytes observed worldwide and at high frequencies in freshwater habitats (Preisig *et al.* 1991). The recent recovery of identical 18S rRNA genotypes from lakes in different parts of the world (Boenigk *et al.* 2005) lends support to the idea that microbial taxa are ubiquitously distributed. There is, however, evidence that very closely related *Spumella* lineages differ in their response to temperature (Boenigk *et al.* 2007). Three different major taxonomic lineages of the *Spumella* morphotype can be distinguished within the chrysophyceaen C-cluster (Andersen *et al.* 1999; Boenigk *et al.* 2005) (Fig. 2a). The biogeographic distribution of these lineages suggests that they are adapted to different climatic conditions: strains affiliated with the C2 subcluster originate mainly from cold environments, i.e. from Antarctica and from high mountain regions; strains belonging to the C1 subcluster are prevalent at temperate sites whereas strains affiliated with the C3 subcluster are found in warm temperate to tropical regions (Fig. 2b). Despite a potentially broader geographic distribution of some members of these subclusters, each has a distinct centre of distribution in different climatic zones.

Consistent with their capacity to grow under potentially suboptimal climatic conditions, we detected representatives of each of the three lineages in the 454 sequence data set. However, we noted substantial seasonal changes in the abundance for all major representatives of these lineages. Interestingly, the seasonal abundance pattern reflected the geographic distribution of the lineages. Spumella-like flagellates were present in Lake Fuschelsee throughout the year (Fig. 2d), but we observed lineage-specific differences with respect to seasonality. Lineage C2, which is dominant in cold environments, was the most abundant one in the early season (Fig. 2c). Lineage C3, described for warm temperate to tropical sites, was most abundant in late summer and early autumn. These results demonstrate that despite a potentially broad distribution of specific protist lineages both, geographical region (i.e. broad climatic conditions) and seasonal variation in environmental factors, are critical for the occurrence at a given site at a given time. Seasonal niche differentiation was not only observed at the level of broader lineages but we also noted a similar phenomenon on the basis of single genotypes. For instance, the two most abundant sequences affiliated with the C1 lineage differ by a single base pair and the genotype, which is abundant in spring is being replaced by the second one later in the year.

### Systematic differential seasonality between rare and abundant taxa conceals the richness of protist communities

**Fig. 3:**
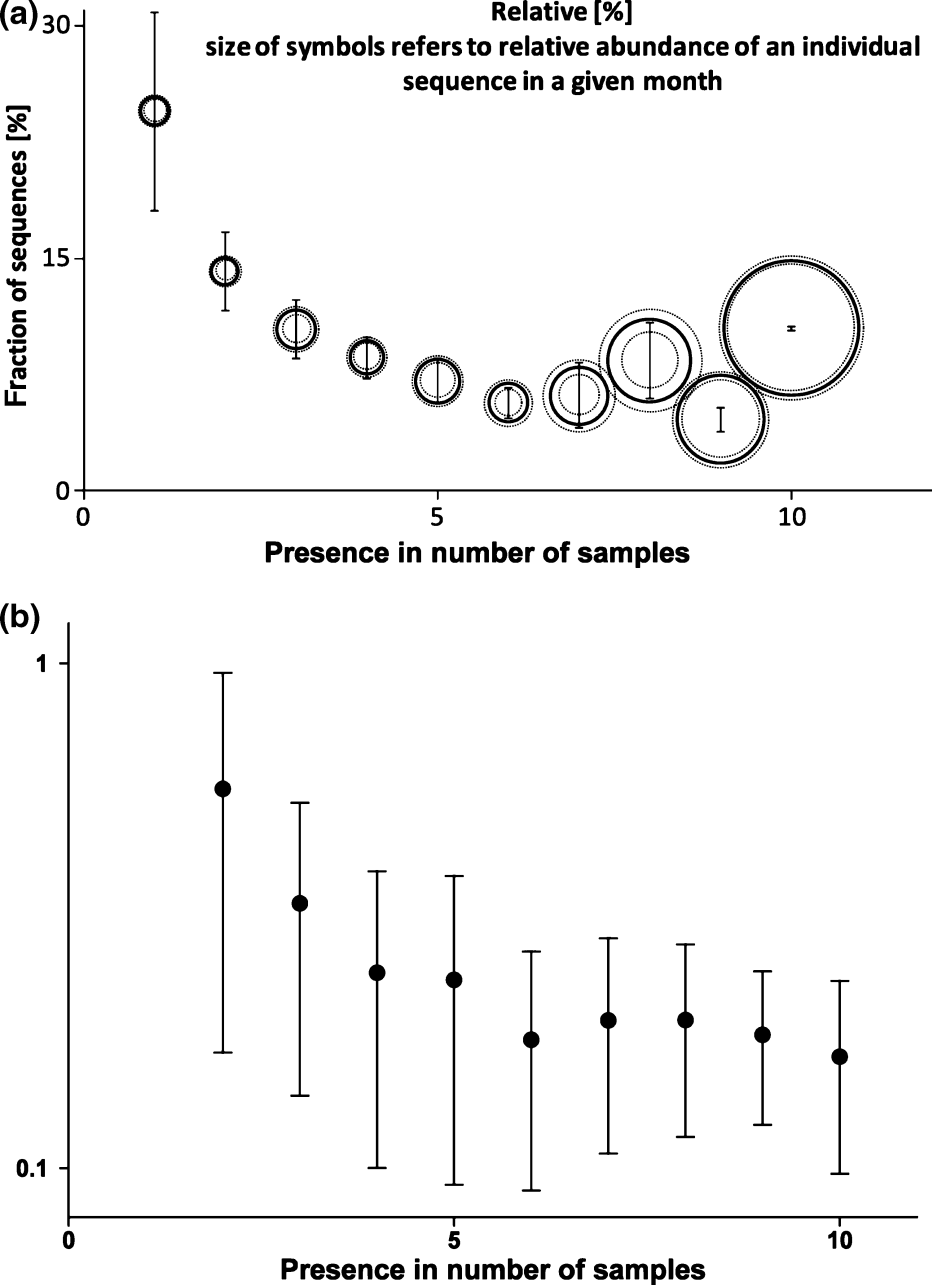
Differential seasonal pattern between rare and abundant taxa. (a) OTU-richness (*y*-axis) and sequence abundance (circle area) of OTUs differing in their seasonal restriction: for each sample OTUs were grouped according to their restriction to either a single sample (*x*= 1) or the presence in a different number of other samples (*x*= 2, …, 10). On average 25% of the OTUs were detected in only one sample. The number of different OTUs decreased with their presence in an increasing number of samples up to a presence in six samples. However, a reverse correlation was observed for OTUs present in more than six samples, i.e. an increasing number of OTUs with increasing number of samples present. Interestingly those OTUs present in most samples were, on average, abundant whereas those OTUs present in few samples were, on an average, rare. (b) We tested whether the pattern observed in (a) is due to a different seasonality between rare and abundant taxa. For this analysis, sequence abundances in the different samples were sorted in decreasing order separately for each OTU. The graph shows the mean and SD of slopes of log-transformed abundances of sequences versus number of samples. On average, the slopes were highest for OTUs restricted to few samples and decreased with the presence of an OTU in an increasing number of samples. Thus, OTUs with a restricted seasonal distribution generally show a stronger increase/decrease of abundance between samples as compared to those OTUs present in most/all samples (for more details see text).

Seasonal changes in frequency have been described in protist communities, but were so far observed only in highly abundant taxa or recorded at higher taxonomic levels (Massana *et al.* 2004; Aguilera *et al.* 2006; Lepère *et al.* 2006; Rynearson *et al.* 2006). The high seasonal turnover in taxa composition in our data set is not a specific phenomenon observed only for few taxa, but is characteristic of all taxa present at low frequency. The dataset was dominated by (i) taxa, which were present in most samples; and (ii) taxa restricted to few samples. Taxa present in an intermediate number of samples were less abundant. Only about 11% of the OTUs were detected in all samples while 25% of the sequences were restricted to a single sample. OTUs, which were detected in multiple samples were more likely to have a high abundance (Fig. 3a) and taxa which were present over a longer seasonal period tended to have more stable population abundances (Pearson’s product–moment: *r*= 0.426; *P* << 0.001; Fig. 3b). These abundant and stable communities are suggestive of a low taxon richness with a moderate seasonal turnover, a pattern that, until recently, has been considered characteristic for microbial eukaryotes (Corliss 1999; Finlay 2001). This pattern is sharply contrasted by the extreme seasonality and pronounced fluctuation of taxon abundance for the majority of taxa. These latter taxa are, however, comparatively rare. The extreme cases of a pronounced seasonality, i.e. month-specific sequences, were occurring only at a very low frequency encompassing on average 1.7% of the reads in a sample. Interestingly, the month-specific sequences accounted for 25% of the total OTU richness. Most importantly, we have no evidence that these low frequency, month-specific sequences are technical artefacts (see Supporting Information). These data indicate that a ‘rare biosphere’ as recognized in prokaryotes by Sogin *et al.* (2006), is also present in the larger and far less abundant picoeukaryotes and undergoes previously unknown dynamic changes.

## Conclusions

In this report we applied massively parallel sequencing of the eukaryote SSU rRNA gene to estimate OTU richness and monitor changes in taxon abundance from a temporal sample collection. Like previous reports on microbial biodiversity (Stoeck *et al.* 2009) we found that the higher sequence coverage substantially increased the estimates of taxonomic diversity when compared to morphological analyses or shallow sequencing of clone libraries. The major surprise of our study was, however, that patterns of protist seasonality systematically differed between rare and abundant taxa and that a large proportion of the biodiversity is detected only in a restricted number of samples: about 50% of the OTUs are detected in no more than three samples (of 10) and 25% are confined to one sample.

Our rarefaction analysis suggested that the total abundance of taxa would be substantially underestimated if we had restricted the analysis to a single sample. While it is not known if the same applies to previous biodiversity estimates, which were based on samples collected at a single time point from a given location (Finlay & Clarke 1999; Green *et al.* 2004; Stoeck *et al.* 2009), it appears highly likely that a broader seasonal sampling would have increased the taxonomic diversity even further.

Our seasonal analysis revealed another interesting insight, which could resolve the long-standing debate about the portioning of species diversity. While the classic view was that species composition does not differ substantially among—physicochemically similar—habitats (Darling *et al.* 2000; Finlay 2002), recent molecular studies challenged this view, by detecting considerable sequence diversity and differentiation among geographically close populations and possibly endemic taxa (Lefranc *et al.* 2005; Rynearson & Armbrust 2005; Rynearson *et al.* 2006; Stoeck *et al.* 2007). Through the use of massively parallel sequencing we were able to analyse a substantially larger number of protist sequences. This extensive data set showed that a small number of highly abundant taxa are present throughout the entire year, while most taxa are rare and highly restricted.

As morphological analyses inherently focus on abundant taxa, a restricted set of taxa is identified in all samples leading to the conclusion that species composition does not differ between habitats. Given that OTUs at low to intermediate frequency form a considerable fraction of the entire samples shallow sequencing will detect a small number of unique sequences. The number of sequenced clones in such studies, however, was generally too low to achieve reliable frequency estimates and it had therefore not been possible to recognize the strong seasonality of the majority of OTUs.

Recent studies indicated that rather than ‘everything’, only generalist taxa may indeed be ubiquitously distributed since they can achieve large population sizes and thereby high dispersal rates (Pither 2007). Our data suggest a systematic difference in the seasonal distribution of abundant and rare taxa, but it is currently unknown whether the abundant taxa with low seasonal turnover in our data set correspond to environmental generalists.

Extending biodiversity studies by temporal sampling could provide support for either hypothesis, ubiquitous dispersal of micro-organisms or the existence of endemic taxa. Without seasonal sampling many rare taxa may be missed due to their strong seasonality and seasonal sampling might lead to finding that indeed everything is everywhere but not at all times. On the other hand, taxa which are temporally highly restricted might be candidates for endemic species present not only in a narrow temporal but also in a restricted spatial range

Hence, we propose that the basic distribution pattern of microbes, including central aspects of the ‘everything is everywhere’ dispute, can only be deciphered by systematically linking a stringent seasonal sampling with extensive sequence coverage. While this request exceeds the capabilities of morphological surveys, molecular approaches based on high-throughput sequencing techniques are promising to meet this claim.

## Supporting Information

**Data S1** Sequences occurring in single samples are not technical artefacts.
